# Diagnostic utility of MRI-based convolutional neural networks in soft tissue sarcomas: a mini-review

**DOI:** 10.3389/fonc.2025.1531781

**Published:** 2025-02-18

**Authors:** Hendrik Voigtländer, Hans-Ulrich Kauczor, Sam Sedaghat

**Affiliations:** Department of Diagnostic and Interventional Radiology, University Hospital Heidelberg, Heidelberg, Germany

**Keywords:** artificial intelligence, soft tissue sarcomas, MRI, CNN, metastasis, recurrence, grade, therapy

## Abstract

**Purpose:**

This review assesses the diagnostic performance of MRI-based convolutional neural networks for identifying and grading soft tissue sarcomas, evaluating therapy responses, and assessing the risk for metastases and recurrences.

**Methods:**

Electronic databases, specifically PubMed/MEDLINE and Google Scholar, were diligently scoured for studies that delved into the intersection of convolutional neural networks, soft tissue sarcomas, and MRI. Three topics were included: 1) differentiating and grading soft tissue sarcomas, 2) assessing therapy response, and 3) predicting metastases and recurrences.

**Results:**

This review included 12 articles. Seven articles investigated the differentiation and grading of soft tissue sarcomas. Sensitivity for that issue ranged from 0.85 to 0.95, specificity from 0,33 to 1, and the area under the curve (AUC) from 0.74 to 0.96. Three articles investigated therapy responses, and two discussed metastasis and recurrence prediction. Only one article out of the five articles above presented accurate diagnostic values. That article examined the prediction of lung metastases and demonstrated a sensitivity of 0.47, a specificity of 0.97, and an AUC of 0.83.

**Conclusion:**

AI applications using CNNs demonstrated robust capabilities in differentiating and grading soft tissue sarcomas using MRI. However, studies on therapy response and prediction of metastases and recurrences are still lacking.

## Introduction

Soft tissue sarcomas account for 1-2% of the overall incidence of adult cancer in Europe (1, 2). The annual incidence of STS varies between 1.8 and 5.0 cases per 100,000 individuals, with a peak occurrence around the age of 60. By 2025, this incidence is projected to increase, primarily attributed to insufficient progress in the prevention, diagnosis, and treatment of these malignancies (3, 4). Moreover, increasing costs of therapy for soft tissue sarcomas are expected, partly due to new drug-based treatments (5, 6). Soft tissue sarcomas are heterogeneous mesenchymal neoplasms with more than 70 histological subtypes (7, 8). Even biopsies can lead to inaccurate results due to this heterogeneity (9). Magnetic resonance imaging (MRI) is the imaging modality of choice for evaluating soft tissue sarcomas with many histological subtypes already been classified using conventional MRI (10–13). There are several classification systems for soft tissue sarcomas. The best-known system is the French Federation Nationale des Centres de Lutte Contre le Cancer (FNCLCC), based on histologic type and subtype features, tumor necrosis, and mitotic activity. It divides soft tissue sarcomas into grades I through III (14, 15). Soft tissue sarcoma staging systems are essential in guiding prognosis and treatment allocation. Precise grading and staging systems can effectively assist with monitoring and preventing local recurrences. However, existing systems do not provide sufficient accuracy for making predictions and are limited by the anatomic stage of the tumor (16). Artificial intelligence (AI) applications using convolutional neural networks (CNNs) offer promising opportunities in many fields of soft tissue sarcoma diagnostics. Capturing and collecting relevant information about pathological changes beyond human visual perception can be a promising application of CNNs in soft tissue sarcoma diagnostics (17, 18). As a consequence, AI might assist less specialized diagnostic centers in making correct diagnoses in the near future (19, 20). AI can also dive far deeper into sophisticated diagnostic methods such as gene sequencing to successfully identify soft tissue sarcomas genetic components (21, 22). Because of its objective and descriptive characteristics, AI can analyze, refine, and quantify medical images. This allows for selecting the most valuable imaging features to analyze clinical information, make differential diagnoses of tumors, and provide accurate guidance for treatment and prognosis (15, 23–27).

In this review, we investigated the potential of CNNs in soft tissue sarcoma diagnostics. For that issue, the diagnostic performance of MRI-based CNNs for differentiating and grading soft tissue sarcomas, evaluating therapy responses and risk for metastases and recurrences were evaluated.

## Materials and methods

### Search strategy

We performed a comprehensive literature review to identify studies assessing the diagnostic performance of convolutional neural networks in magnetic resonance imaging of soft tissue sarcomas. Therefore, PubMed/MEDLINE and Google Scholar were systematically searched using selected keywords. These selected keywords and terms included “soft tissue sarcoma”, “machine learning”, “deep learning”, “artificial intelligence”, “convolutional neural network” and “MRI”. We applied multiple combinations of these keywords with appropriate Boolean operators (OR/AND) to each online database.

### Inclusion and exclusion criteria

All observational studies on three areas of soft tissue sarcoma diagnostics were included: 1) differentiating and grading soft tissue tumors, 2) predicting metastases and recurrences, and 3) assessing therapy response. The following studies were excluded: (1) other types of studies than observational (including case reports/series, editorials, comments, correspondence, guideline, experimental, and interventional studies, as well as meta-analyses, systematic and narrative reviews); (2) grey literature or literature produced outside of the traditional academic publishing channels; (3) articles lacking available full texts in English; (4) articles using other imaging modalities than MRI; (5) animal studies; (6) studies without values on diagnostic accuracy; (7) studies not falling under the three included topics (differentiating/grading, therapy response, prediction of metastasis/recurrence).

### Literature search

Eighty-one publications were initially identified. After eliminating 29 duplicate studies, 52 articles were considered for title/abstract screening. After this screening, 38 studies advanced to the full-text examination phase. Following the full-text review, 26 articles were excluded due to not falling into the inclusion criteria, leaving 12 articles that addressed the research questions, met the inclusion criteria, and were therefore included in the final study (**Table 1**).

**Table 1:** Overview of the included studies with study design and results.

Subject area	Author	Year	n	Aim	number of CNNs used	Best performed CNN	AUC	Sensitivity	Specificity	Accuracy
**Differentiating and grading**	Dai et al. (28)	2021	172	Differentiation of soft tissue sarcomas and atypical lipomas	4	mp ResNet 50	0.96	0.85	–	0.87
	Gitto et al. (29)	2023	150	Differentiation lipomas and atypical lipomatous tumors	3	RF	0.74	0.92	0.33	–
	Malinauskaite et al. (30)	2020	38	Differentiation of lipomas and liposarcomas	4	SVM	0.926	0.88	1.0	0.927
	Navarro et al. (31)	2023	158	Grading of soft tissue sarcomas	15	DenseNet 161	0.75	0.91	0.4	0.83
	Peeken et al. (32)	2019	225	Grading of soft tissue sarcomas	7	Radiomics combined LASSO-based	0.84	0.90	0.5	0.83
	Xu et al. (33)	2020	105	Differentiation of soft tissue sarcomas according to malignancy grade	11	LASSO + RF	0.922	0.882	0.944	0.9143
	Yang et al. (34)	2022	127	Prediction of MDM2-Gene amplification to differentiate liposarcomas and lipomas	6	ResNET 50	0.95	0.95	0.89	0.9211
**Therapy response**	Blackledge et al. (35)	2019	18	Response assessment	8	RF	–	–	–	0.981
	Gao et al. (36)	2021	30	Assessment of therapy response to radiotherapy	6	VGG 19	–	–	–	0.833
	Peeken et al. (37)	2021	156	Response assessment in neoadjuvant therapy	4	RF-based delta combined	0.79	–	–	–
**Predicting metastasis and recurrence**	Liang et al. (38)	2022	351	Prediction of lung metastases	3	DLRN based on ResNet 34 in combination with mRMR+LASSO+SVM+SMOTE	0.833	0.474	0.972	0.897
	Liu, S. et al. (39)	2021	113	Prediction of recurrences	2	DLRN 2-based ResNet 34	0.96	–	–	–

RF, Random forest; VGG 19, Visual Geometry Group 19 Layer; mp, multiparametric; mRMR, minimum redundancy maximum relevance; LASSO, least absolute shrinkage and selection operator; SVM, support vector machine; SMOTE, synthetic minority over-sampling technique; DLRN, deep learning radiomics nomogram; ERT, extremely randomized trees; RFE, recursive feature elimination technique; STT, SMOTETomek; SVM, support vector machine; CHMFL, constrained hierarchical multi-modality feature learning; 3DMCL, 3D deep multi-modality collaborative learning. Model C + R, clinical and radiomics.

## Results

### Differentiating and grading soft tissue sarcomas

Dai et al. used a ResNet 50 model to differentiate between soft tissue sarcomas and atypical lipomas, achieving an AUC of 0.96, a sensitivity of 0.85, and an accuracy of 0.87 (28). Gitto et al. applied a Random Forest (RF) model to distinguish lipomas from atypical lipomatous tumors, resulting in an AUC of 0.74, high sensitivity (0.92), but low specificity (0.33). Gitto et al. found no significant difference between the AI’s performance and a radiologist’s, with the AI exhibiting a sensitivity of 0.92 compared to the radiologist’s 0.88 and a specificity of 0.33 versus the radiologist’s 0.54 (p=0.474) (29). Malinauskaite et al. utilized a Support Vector Machine (SVM) to differentiate between lipomas and liposarcomas, with an AUC of 0.926, sensitivity of 0.88, specificity of 1.0, and an accuracy of 0.927. Malinuskaite et al. found that the best-performing AI model out of their four CNNs surpassed the results of three radiologists with varying experience (10, 5, and 2 years specializing in musculoskeletal radiology). Specifically, the AI’s AUC was 0.926 compared to the radiologists’ 0.804, with a sensitivity of 0.88 versus 0.769, specificity of 1.0 against 0.84, and accuracy of 92.7% compared to 81.6% (30). Navarro et al. and Peeken et al. focused on grading soft tissue sarcomas using DenseNet 161 and LASSO-based models, achieving AUCs of 0.75 and 0.84, respectively, with similar sensitivities (0.91 and 0.90) and specificities (0.4 and 0.5) (31, 32). Xu et al. and Yang et al. differentiated soft tissue sarcomas according to malignancy grade and predicted MDM2-Gene amplification, respectively, achieving AUCs of 0.922 and 0.95, with high sensitivity and specificity values (33, 34).

### Therapy response

Blackledge et al. achieved an accuracy of 0.981 in response assessment using an RF model (35). Gao et al. reported an accuracy of 0.833 in assessing therapy response to radiotherapy using a VGG 19 model (36). Peeken et al. used an RF-based delta model combined with other metrics to assess neoadjuvant therapy response, with an AUC of 0.79 (37).

### Predicting metastasis and recurrence

Two studies focused on predicting metastases and recurrences. Liang et al. used a complex DLRN model based on ResNet 34 combined with multiple other algorithms (mRMR+LASSO+SVM+SMOTE) to predict lung metastases, achieving an AUC of 0.833, with sensitivity and specificity values of 0.474 and 0.972, respectively (38). Liu, S. et al. (2021) predicted recurrences using a DLRN 2-based ResNet 34 model, achieving a high AUC of 0.96 (39).

## Discussion

This review investigates the potential of MRI-based CNNs for identifying and grading soft tissue sarcomas, evaluating their therapy responses, and assessing the risk for metastases and recurrences.

As soft tissue sarcomas comprise a rare and heterogeneous group of malignancies, conventional diagnostic features assessing soft tissue sarcomas themselves, therapy responses, and potential risk factors for metastases and recurrences are rare. Although soft tissue sarcomas present some characteristic findings on MRI (8, 15, 23, 40–42), these characteristics are still insufficient for an overall differentiation/grading and risk stratification of soft tissue sarcomas in imaging. Integrating AI in soft tissue sarcoma diagnostics offers a promising avenue for disease management, diagnostics, and prognosis. However, as with any pioneering methodology, the implementation of AI comes with challenges. One significant issue is the small sample size observed in most studies. Peeken et al. circumvented this by orchestrating multicentric studies to bolster patient numbers (32, 37, 43). Despite such attempts, the maximum number of patients recorded in any analyzed study did not surpass 351. Meanwhile, Gao et al. adopted a different approach, producing over 15,000 synthetic images through oversampling (36). The data imbalance poses a second critical challenge. Liu X. et al. formulated a unique SRS strategy that involved two-step data splitting to enhance the balance between the training and testing datasets (44). However, this methodology led to overlaps, thereby compromising the integrity of the “true” test numbers. CNNs remain at the heart of the issue. Often perceived as black boxes, the proper CNN selection is paramount for effective results (45–48). For instance, while ResNet layers have been found to be an application, excessive layering can usher in issues like vanishing gradients, where the learning network becomes heavily dependent on initial weights, causing a regression in learning (49). Another persistent challenge is using retrospective data, which, though readily available, brings forth issues like the inability to alter past MRI settings, susceptibility to errors, and bias (50). The absence of open metadata, coupled with variable metrics and cut-offs, obstructs the comparison of study results. Guaranteeing transparency and reproducibility necessitates detailed reports on metrics, statistical hypotheses, and specific cut-offs (51).

Despite these challenges, milestones have been achieved. Yang et al., for instance, demonstrated that MDM2 gene amplification could be gleaned from image data alone (34). While certain inaccuracies like misestimating tumor grading persist, as evidenced by Xu et al.’s 26.9% upgrade rate, harnessing ample data and prudent feature selection can deliver reliable outcomes (33).

Another critical limitation is the scalability and high cost of these solutions, with implementation costs estimated to reach up to $1 million, depending on factors such as data acquisition, infrastructure, and regulatory compliance (52, 53). High computational demands and infrastructure requirements hinder adoption, particularly in low-resource settings (19–21, 52, 53). Additionally, the environmental impact of AI training processes, which contribute to increased CO2 emissions, cannot be overlooked (54, 55).

This review revealed that most studies in soft tissue sarcoma diagnostics have focused on grading and differentiating these tumors. However, research on therapy response and risk stratification for metastases and recurrences remains limited. While the overall diagnostic performance of CNN-based applications for grading and differentiating soft tissue sarcomas is relatively high, the included studies demonstrated significant variation in specificity compared to sensitivity, with some studies reporting specificities as low as 0.33. Therefore, future CNN-based applications should aim to improve specificity while maintaining high sensitivity. Yet, with continuous refinement, AI’s potential in soft tissue sarcomas and medicine, in general, is undeniable.

The future might very well behold a time when we can ascertain the grading of soft tissue sarcomas without necessitating punctures, which could lead to quicker and more effective therapy and in total lower the cost of the treatment.

The present study has several limitations. The included studies showed heterogeneous study designs, objectives, and sample sizes, making comparisons challenging. Additionally, some of the included studies had small sample sizes, which could have affected the diagnostic performance of the AI applications included. Also, most studies did not investigate confounding factors, which might have influenced outcomes.

## Conclusion

Applications of convolutional neural networks (CNNs) demonstrate significant potential for differentiating and grading soft tissue sarcomas using MRI. However, there remains a gap in research on evaluating therapy responses and predicting metastases and recurrences, underscoring the need for further investigation in these critical areas. This study highlights the potential of AI to enable precise, non-invasive diagnostic methods for soft tissue sarcomas, reducing the reliance on invasive procedures. In the future, AI could become a valuable tool for effective treatment planning, ultimately improving patient outcomes and optimizing healthcare resources.
